# Chemical volatiles present in cotton gin trash: A by-product of cotton processing

**DOI:** 10.1371/journal.pone.0222146

**Published:** 2019-09-18

**Authors:** Mary A. Egbuta, Shane McIntosh, Daniel L. E. Waters, Tony Vancov, Lei Liu

**Affiliations:** 1 Southern Cross Plant Science, Southern Cross University, Lismore, New South Wales, Australia; 2 New South Wales Department of Primary Industries, Wollongbar, New South Wales, Australia; 3 ARC ITTC for Functional Grains, Charles Sturt University, Wagga, Wagga, New South Wales, Australia; Higher Institute of Applied Sciences and Technology of Gabes University of Gabes, TUNISIA

## Abstract

Cotton gin trash (CGT), a waste product of cotton gins, make up about 10% of each bale of cotton bolls ginned. The current study investigates high value volatile compounds in CGT to add value to this by-product. The volatile compounds in CGT and different parts of the cotton plant were extracted using various methods, identified by gas chromatography-mass spectrometry (GC-MS) or nuclear magnetic resonance (NMR) spectroscopy, and then quantified by gas chromatography-flame ionisation detection (GC-FID) against available standards. Terpenoids including monoterpenoids and sesquiterpenoids were found to be the most abundant, making up 64.66% (area under peak) of total volatiles extracted by hydro-distillation. The major extractable terpenoids in CGT were α-pinene (13.69–23.05 μg/g), β-caryophyllene (3.99–74.32 μg/g), α-humulene (2.00–25.71 μg/g), caryophyllene oxide (41.50–102.08 μg/g) and β-bisabolol (40.05–137.32 μg/g). Recoveries varied between different extraction methods. The terpenoids were found to be more abundant in the calyx (659.12 μg/g) and leaves (627.72 μg/g) than in stalks (112.97 μg/g) and stems (24.24 μg/g) of the cotton plant, indicating the possible biological origin of CGT volatiles. This study is the first to identify and quantify the different terpenoids present in CGT and significantly, β-bisabolol, an abundant compound (sesquiterpene alcohol) which may have valuable biological prospects. These findings therefore contribute to identifying alternative management strategies and uses of CGT.

## Introduction

Cotton gin trash (CGT) is the waste product generated from cotton ginning, a process whereby mature cotton bolls are separated into different components such as seeds and fibres, which are used as raw materials for production of end products such as cotton seed oil and fabric [1]. Comprising mainly leaves, sticks, calyx (burrs), lint and soil, CGT makes up about 10% of each bale of cotton ginned, and therefore, results in several million tons of CGT generated yearly [1,2]. Normal disposal of cotton gin trash has been by incineration, landfilling and composting, and in some cases, it is used as feed supplements for livestock [3,4]. Recently, CGT has also been exploited as an alternative source of sugars [5] for conversion into bioethanol [6–9] since it contains considerable amount of fibre and has been piled up closed to the ginning factories. Although the exploitation of CGT for ethanol is possible, this is not considered a commercially viable pathway for utilisation of the trash. Therefore, it is necessary to investigate other possible uses of CGT in order to add value to this by-product.

Volatile chemicals are typical secondary metabolites of many plants including cotton [10,11]. These volatile compounds, mainly terpenoids, are stored in plants and released when needed, or can be synthesised at the time of need [12,13]. Monoterpenoids and sesquiterpenoids are the most common terpenoids in cotton and are distributed in different parts of the plant including leaves, boll, calyx, stem, stalks and roots [14–16]. Terpenoids found in cotton include pinene, myrcene, caryophyllene, humulene, caryophyllene oxide and bisabolol etc. [17–20]. Some of these terpenoids are known to be of potentially high value due to their biological activities which may have applications in agricultural and pharmaceutical industries [21–23]. The presence of these high value terpenoids distributed throughout the cotton plant therefore triggers some motive to exploit CGT as a potential source of these volatile chemicals. This assumption has been partially confirmed by Agblevor et al. (2003), who reported the proximate composition of CGT and different groups of compounds such as total carbohydrates and extractives, although the individual extractives were not characterised [23].

Generally, determination of the composition of plant volatiles can be achieved by extracting the compounds from various parts of the plant. Extraction procedures are dependent on the nature (matrix properties) of the plant and its parts, and differ by temperature, extraction solvent, time and pressure [24,25]. Methods such as hydro-distillation, headspace, soxhlet, masceration, microwave assisted extraction, ultrasound assisted extraction, enzyme assisted extraction and supercritical fluid extraction are examples of effective techniques for extracting bioactive compounds from plant materials [24,26–29]. These techniques have been implemented in extraction of volatile compounds depending on the nature of the target compounds in different plants, including isolation from cotton tissues. Evaluation of extractives from the cotton plant by hydro-distillation [20], particularly from aerial parts of the plants, identified terpenoids such as pinene, limonene, caryophyllene and humulene. The organic solvents, hexane and pentane were used by Opitz et al. (2008) to determine the concentration in cotton leaves of some terpenoids including α and β –pinene, myrcene, ocimene, caryophyllene, humulene and β-bisabolol [19]. Recovery of these terpenoids demonstrated the effectiveness of these methods to determine phytochemicals of the cotton plant. With regards to CGT, research concerning recovery of extractives is seldom reported, though, evaluation of total extractives by Agblevor et al. (2003) was done by Soxhlet extraction using 95% ethanol but no particular compound was identified or quantified [23].

Although phytochemical study of cotton suggests the plant is a reservoir of extractives, particularly terpenoids, information on the complete volatile composition of CGT is still unclear. In this study, different extraction and analytical methods were implemented to achieve recovery, identification and quantification of terpenoids present in the trash. Methods implemented for extraction of compounds from CGT are well established for extracting a wide range of chemicals from plant materials [24,30]. These methods are also suitable for use at an industrial scale. Postharvest trash (PHT) or the cotton plant material left standing in the field after harvesting also represents a significant biomass feedstock, hence, PHT was comparatively investigated for volatile extractives. The different components of CGT and PHT were also analysed for terpenoids composition in order to determine the most valuable component. Ultimately this information may underpin a more lucrative product diversification for the cotton industry.

## Materials and methods

### Reagents and analytical standards

Analytical standards were procured from Sigma Aldrich, Australia and included (+)-α-pinene (#80605), (-)-β-pinene (#80609), α-humulene (#12448), β-humulene (#53676), (-)-α-bisabolol (#95426), (-)-caryophyllene oxide (#91034) and (-)-*trans*-caryophyllene (#75541). Organic solvents n-hexane, methanol, ethanol, dichloromethane, diethyl ether and diethyl acetate were of HPLC grade and bought from Scharlau Australia.

### Sample preparation

CGT was kindly supplied by Namoi Cotton Co-operative (Yarraman Gin, NSW). All material was air-dried at 40 °C for 48 h. To achieve better uniformity and further size reduction, samples were subject to a 60 sec pulse in a pulveriser (Labtechnics Pulveriser, WA, Australia). Samples were stored at room temperature prior to extraction. Dried post–harvest trash (PHT) samples were first separated into the different components of stalks, stems, leaves and calyx (burr) prior to grinding using a Mixer mill 301 (Retsch GmbH, Germany).

### Hydro-distillation and gas chromatography-mass spectrometry (GC-MS)

#### Preliminary distillation

Preliminary hydro-distillation were performed to determine an optimised duration for the extraction procedure. CGT sample (300 g) was distilled in 2,700 mL milli-Q water sequentially for 6 h, 12 h and 18 h and extracted oil collected for the three time durations. Recovered oil was extracted with hexane and the extract analysed for terpenoids by gas chromatography-mass spectroscopy. The methods finally used for extraction, identification and semi-quantification of the volatiles from the trash samples are explained in the following sections.

#### Hydro-distillation of CGT

For all hydro-distillation, 300 g of CGT sample was submerged in 2700 mL milli-Q water and boiled for six hours after mixing in a 5,000 mL round bottom flask connected to a chiller and essential oil collected in a graduated collection tube. Following distillation, the volume of oil was measured using a graduated collection tube. The collection tube was washed with acetone and combined with collected oil was dried in a fume hood overnight. The recovered extracted oil was mixed with 3 mL milli-Q water and 5 mL hexane, shaken vigorously and centrifuged at 3,000 rpm for 3 mins. The hexane fraction was collected in a 25 mL volumetric flask and the extraction procedure repeated twice with equal volumes of hexane. Total extract in the volumetric flasks were made up to 25 mL and transferred to clean, pre-weighed 40 mL vials. Extracts were stored at 4 °C until further analysis.

Identification and semi-quantification was performed on an Agilent 6890A GC instrument equipped with a ZB-5 capillary column (Phenomenex) of dimensions 300 mm length, × 0.53 mm internal diameter (I.D) × 1.50 μm film thickness. Samples were injected in the split mode (split ratio of 1:25) under the following conditions: injection port set at 280 °C, oven temperature held at 50 °C for 1 min, then programmed at a rate of 8 °C/min to 300 °C, helium carrier gas at a flow rate of 1.2 mL/min. An Agilent 5973 network mass selective detector was used as the detector and operated in scan mode, with a scanning mass range of 35 to 300 atomic mass unit (amu) at 5.19 scans/sec. Electron ionisation (EI) for the mass selective detector was 70eV and a solvent delay time of 6 mins was maintained when no spectra was collected. Volatile compounds in CGT extracts were identified by comparing mass spectrum with GC-MS library Wiley 275.L database and their relative abundance were compared using the peak area in the total ion chromatogram (TIC).

### Isolation and identification of unknown sesquiterpenoid

CGT oil obtained by hydro-distillation was fractionated using high-performance liquid chromatography (prep-HPLC, Agilent Technologies 1260) equipped with an ultraviolet (UV) detector and fraction collector. The CGT oil (400 μl) was mixed with methanol (600μl) before injection. Separation of fractions was performed using a Luna C_18_ column (150 mm x 21.20 mm, 55 μm, Phenomenex Co., USA) at a flow rate of 20 mL/min of mobile phase with methanol + 0.05% trifluoroacetic acid (TFA) and water +0.05% TFA. A linear gradient elution mode was maintained with mobile phase set at 0 min for 60% methanol, 2 min for 60% methanol, 8 min for 100% methanol, 22 min for 100% methanol, 25 min for 60% methanol and 30 min for 60% methanol. The UV detection was at 210, 280 and 360 nm. A total of four injections of 250 μl each were done with fractions collected between 4 and 18 min at an interval of 0.24 min.

The purity of each fraction was checked using the GC-MS method described above and liquid chromatography-mass spectrometry (LC-MS, Agilent Technologies 1260), equipped with a vacuum degasser, binary pump and auto injector, diode array detector (DAD, 1260) and quadrupole mass detector (MSD, 6120). An Agilent eclipse plus C18 RRHD column with specifications of 1.8 μm, 2.1 x 50 mm was used. The mobile phase was composed of water and 0.005% TFA and acetonitrile and 0.005% TFA and the flow rate was at 0.3 mL/min. Samples were analysed using a linear elution gradient with acetonitrile, 10% at 0 min, 99% at 10 min, 99% at 11.5 min, 10% at 13 min and 10% at 15 min. Fractions collected from prep-HPLC were directly injected for LC-MS analysis and injection volume was 0.1μl. The mass selective detection (MSD) was carried out in electrospray ionisation (ESI) mode using the following parameters: drying gas flow, 12.0 L/min; nebulizer pressure, 35 psig; drying gas temperature, 350 °C; capillary voltage, 3000 V (positive) and a scan mass range of 100 *m/z* to 1200 *m/z*.

Fractions containing unknown sesquiterpenoids were dried under a stream of nitrogen gas, dissolved in methanol and further purified using the same prep-HPLC described above fitted with a semi-preparative HPLC column (Luna C_18_ column, 250 x 10mm, i.d 55μm, Phenomenex Co., USA). The flow rate was at 4 mL/min with a linear gradient of methanol 0 mins-85%, 2 mins-85%, 20 mins-93%, 20.5 mins-100%, 21.1 mins-100%, 22 mins-85% and 30 mins-85% methanol. The fractions were collected between 10.70 and 15.00 mins at time slice of 0.10 min and their purity was checked using the GC-MS and LC-MS method described above. The confirmed pure fractions were dried under a stream of nitrogen gas and subjected to nuclear magnetic resonance (NMR) spectroscopy analysis.

The chemical structure and identity of the isolated compound was confirmed by ^1^H-NMR and ^13^C-NMR and 2D (heteronuclear multiple bond correlations (HMBC), homonuclear correlation spectroscopy (COSY) and rotating-frame overhauser spectroscopy (ROESY)) NMR using a Bruker 800MHz NMR with the isolated compound dissolved in deuterated chloroform (CDCl_3_). Optical rotation measurement was conducted on a JASCO P-1020 Polarimeter to confirm the stereochemistry of the compound isolated.

### Comparison of organic solvent extractions

The organic solvents hexane, methanol (MeOH), dicholoromethane (DCM), diethyl ether (DEE), ethanol and ethyl acetate (EA) were used to extract volatile compounds from 1 g of CGT samples. Parallel extraction of compounds using each organic solvent was done in triplicate. One gram of CGT was weighed into 22 mL vials and 3 mL of each respective extraction solvent added. The mixture was sonicated for 15 min in a sonication bath (Soniclean) and centrifuged using a benchtop centrifuge (Sigma 2–5 10134 centrifuge) at 3,000 rpm for 3 mins. The supernatant (extraction solvent) containing extracts from the CGT sample was transferred into a clean 10 mL volumetric flask. The extraction procedure using 3 mL of solvent was repeated twice and the supernatant collected in a volumetric flask and the volume made up to 10 mL with addition of the respective solvent. Each type of solvent extract was transferred into clean, pre-weighed 22 mL vials and stored at 4 °C until further analysis. One mL aliquots were collected from all extracts into clear 2 mL screw-cap HPLC vials and kept at 4 °C with bulk extracts for further analysis.

PHT components were weighed (1 g) into clean 22 mL vials and 10 mL s of extraction solvent, methanol and hexane added to the samples. Extraction was done in duplicate. Samples were sonicated for 30 mins and left overnight to allow maceration of samples. The following day, samples were vigorously shaken by hand and centrifuged at 3,000 rpm for 3 mins. The supernatant (extraction solvent) was collected into clean, pre-weighed 22 mL vials and 1 mL aliquots collected for further analysis. All extracts were stored at 4 °C until further analysis.

Volatiles were extracted from a larger CGT sample using methanol and hexane. In each 500 mL conical flask, 40 g of CGT was mixed with 200 mL extraction solvent and sonicated for 1 h. After sonication, the respective CGT and extraction solvent mixtures were filtered through a Whatmann Grade 4 filter paper. At this stage, 1 mL aliquots were collected from the respective extraction solvents for further analysis.

### Quantification by gas chromatography-flame ionisation detection (GC-FID)

A Hewlett Packard 6890 series GC instrument equipped with a BPX-5 capillary column (SGE Analytical Science) (50 mm x 0.22 mm x 1 μm film thickness) fitted with a flame ionisation detector was used. Samples were injected in split mode (split ratio of 1:25) under the following conditions: injection port set at 280 °C, oven temperature held at 50 °C for 1 min, then programmed at a rate of 8 °C/min to 300 °C, helium carrier gas at a flow rate of 1.2 mL /min. Samples were analysed alongside analytical standards of known concentration (β-caryophyllene 4.5 mg/g, α-bisabolol 3.6 mg/g, caryophyllene oxide 1.0 mg/g, α-pinene 4.4 mg/g, β-pinene 4.5 mg/g and α–humulene 2.1 mg/g) and concentration of volatiles calculated using equations derived from standard calibration curve plotted for dilutions of analytical standards.

### Data analysis

Data generated from each experiment was analysed using MSD chemstation data analysis software for GC-MS and LC-MS. Microsoft Excel, 2013 was used to calculate concentration, mean values and standard deviation and percentage composition of identified chemical volatiles. GenStat 64-bit Release 18.1 (18^th^ edition), was used to calculate analysis of variance (ANOVA) and determine significant difference (P≤0.05) using the Duncan’s multiple range test. NMR spectroscopy data were analysed using Bruker’s TopSpin^™^ software.

## Results

### GC-MS analysis of volatiles from CGT extract by hydro-distillation

Gas chromatography-mass spectrometry analysis of extracts obtained from hydro-distilled CGT oil showed different volatile chemicals. Preliminary experiments to determine the appropriate duration (6 h, 12 h or 18 h) for maximal extraction of volatile compounds from CGT by hydro-distillation indicated that most of the terpenoids in CGT were extracted after 6 h of distillation (S1 Table). After the first 6 h of hydro-distillation, 91.2% of the total extractible oil was obtained from CGT. Extending the hydro-distillation duration only slightly increased the yields by 7.0% and 1.8% at 12 h and 18 h, respectively. Likewise, most of the terpenoids were present in the CGT extract oil obtained from the 6 h hydro-distillation, particularly the monoterpenoids (α-pinene, myrcene and α-copaene) and sesquiterpenoids (β-caryophyllene, α-humulene and α-bisabolol). Hydro-distillation for 6 h recovered between 89% and 97% of the larger sesquiterpenoid compounds, therefore, the duration was subsequently used in all ensuing hydro-distillation extractions.

**Fig 1:**
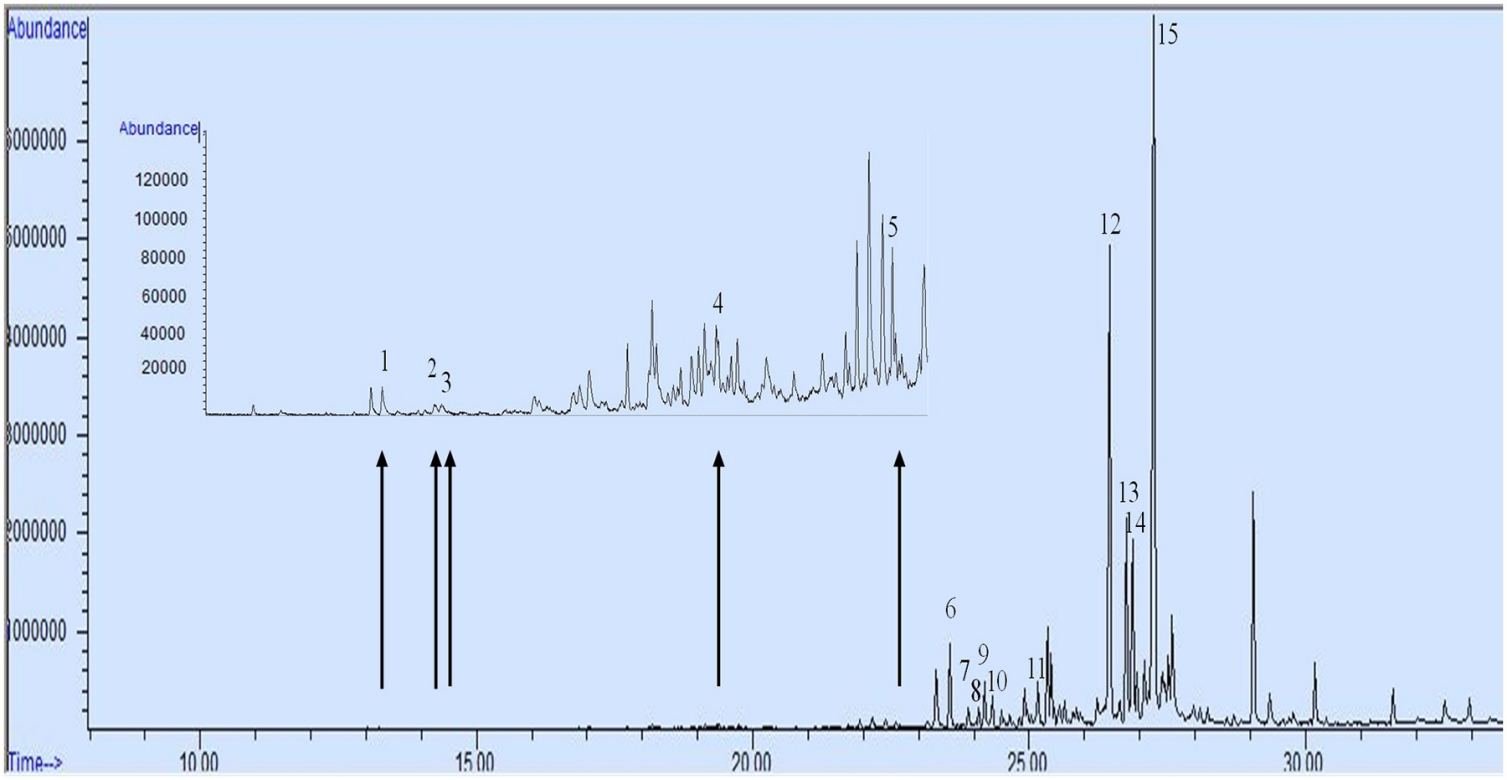
Total ion chromatogram of CGT extract from hydro-distillation indicating the different terpenoids identified and semi-quantified. Peak labelled with numbers on top are terpenoids (% area of total area under peaks): (1) α-pinene (0.04), (2) myrcene (0.02), (3) β-pinene (0.01), (4) safranal (0.03), (5) α-copaene (0.1), (6) β-caryophyllene (2.4), (7) β-santalene (0.5), (8) α-curcumene (0.4), (9) α-humulene (1.2), (10) β-farnesene (0.7), (11) nerolidol (1.2), (12) caryophyllene oxide (15.5), (13) gossonorol (5.7), (14) humulene epoxide II (5.3) and (15) β-bisabolol (28.9).

Detection of the major terpenoids (Fig 1) showed retention times of between 13.23 to 22.61 min for monoterpenoids whereas, the larger sesquiterpenoids followed after with retention times between 23.57 and 27.25 min. Using the sum of all peak areas identified in the extract, the percentage composition of terpenoids was determined (Fig 1). The most abundant compound in CGT oil from hydro-distillation was tentatively identified as β-bisabolol (28.9%) based on GC-MS library with up to 98% quality match. Other major peaks in the chromatogram which are not labelled represent hydrocarbons and fatty acids (e.g. hexadecanoic acid, pentadecanone and farnesyl acetone) also extracted from CGT hydro-distilled oil (S2 Table).

Terpenoids identified in this study from the oil extracts of hydro-distilled CGT samples comprised of 64.7% (area under peaks) total terpenoids, consisting of about 0.1% and 64.5% monoterpenoids and sesquiterpenoids, respectively. The other non-terpenoid volatiles constituted 35.3% of total volatiles identified in the CGT samples.

### Confirmation of β–bisabolol by NMR spectroscopy

In order to confirm the identity of the most abundant volatile in the extract of CGT which was tentatively identified as β-bisabolol by GC-MS analysis, isolation by prep-HPLC and semi-prep HPLC (S1 Fig) followed by NMR spectroscopy was performed. The isolation and analysis were performed as analytical standard of β-bisabolol was not commercially available.

**Fig 2:**
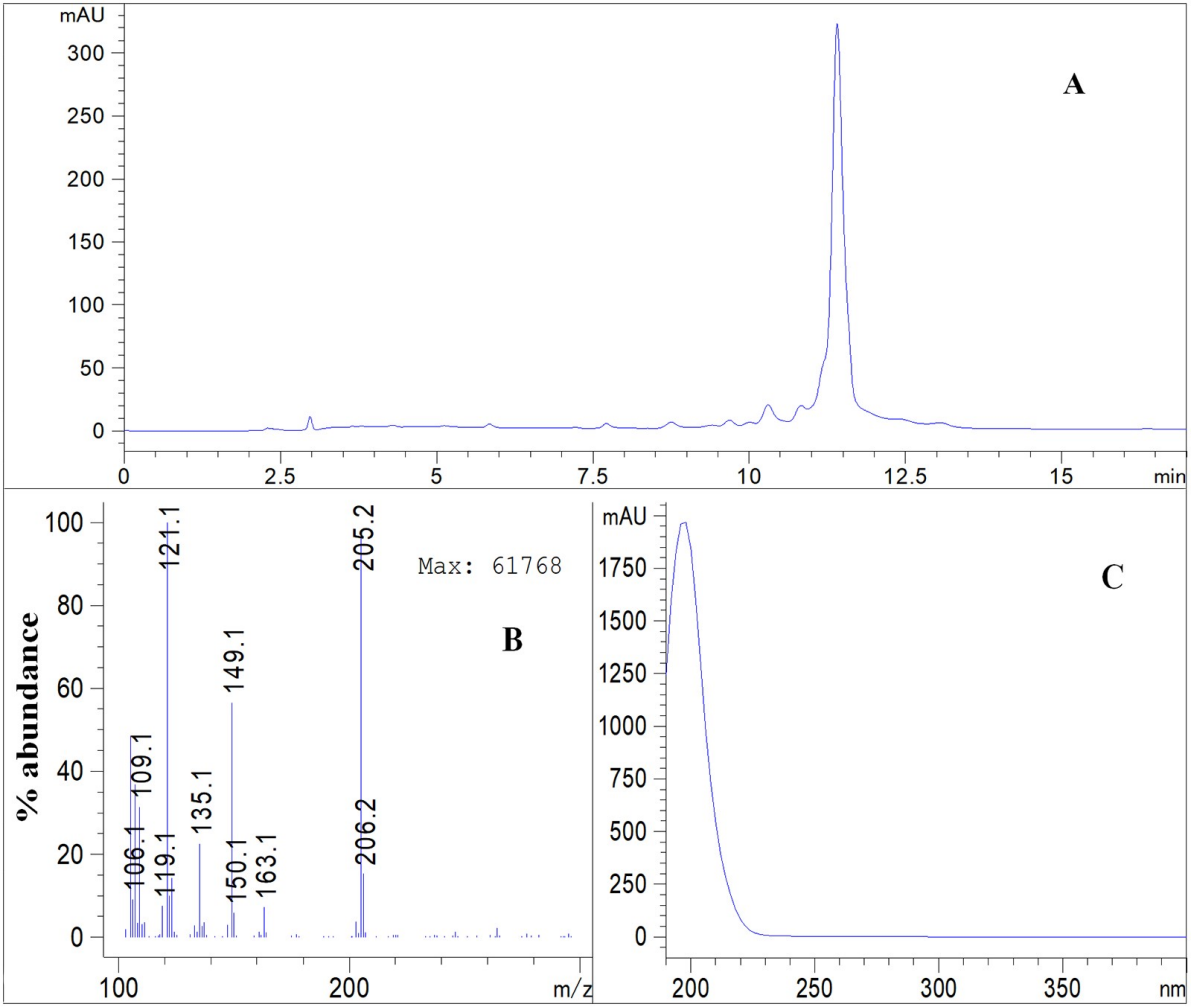
Liquid chromatography total ion chromatogram (A), mass spectrum (B) and UV spectrum (C) of isolated β-bisabolol.

**Fig 3:**
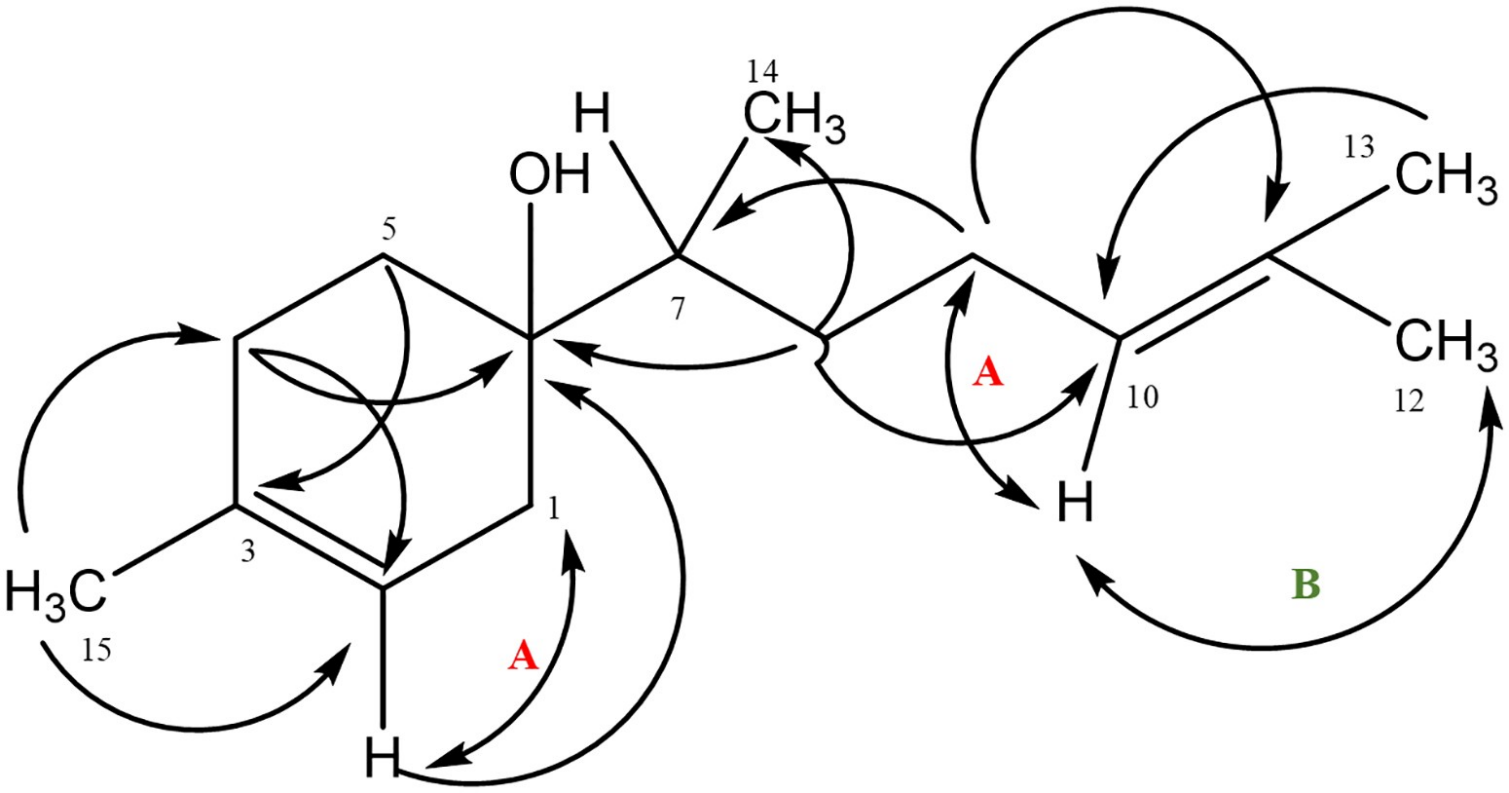
Chemical structure of isolated β-bisabolol showing key HMBC, COSY (A) and ROESY (B) correlations.

Data obtained from LC-MS analysis of the isolated compound represented in Fig 2 show total ion chromatogram, mass and ultraviolet (UV) spectrum of the compound 6-epi-β-bisabolol (6R, 7S) identified by NMR spectroscopy and optical rotation data _D_ = -68.2° at 28.7°C (c = 0.0733mg/mL in CHCl_3_). The chemical structure was elucidated from NMR spectroscopy (Fig 3) with key HMBC, COSY and ROESY correlations. Peak assignments for [^1^H] and [^13^C] NMR spectroscopy of the identified β-bisabolol are presented in Table 1.

**Table 1:** [^1^H] and [^13^C] NMR spectroscopy peak assignments of the terpenoid β-bisabolol isolated from CGT hydro-distilled oil.

C	Carbon shift (δ_C_)	Proton shift (δ_H_)
1	34.4	(1.86, 2.17)
2	118.6	5.30 (s)
3	134.2	
4	27.2	(1.94, 2.17) m
5	31.2	(1.59, 1.62) m
6	72.4	1.62 (s)
7	42.2	1.45 (m)
8	31.1	(1.05, 1.69) m
9	26.8	(1.91, 2.10) m
10	125.0	5.12 (t)
11	131.6	
12	25.9	1.69 (s)
13	17.7	1.61 (s)
14	13.8	0.92 (d)
15	23.5	1.67 (s)

### Quantification of terpenoids in CGT by hydro-distillation and different organic solvent extractions

**Fig 4:**
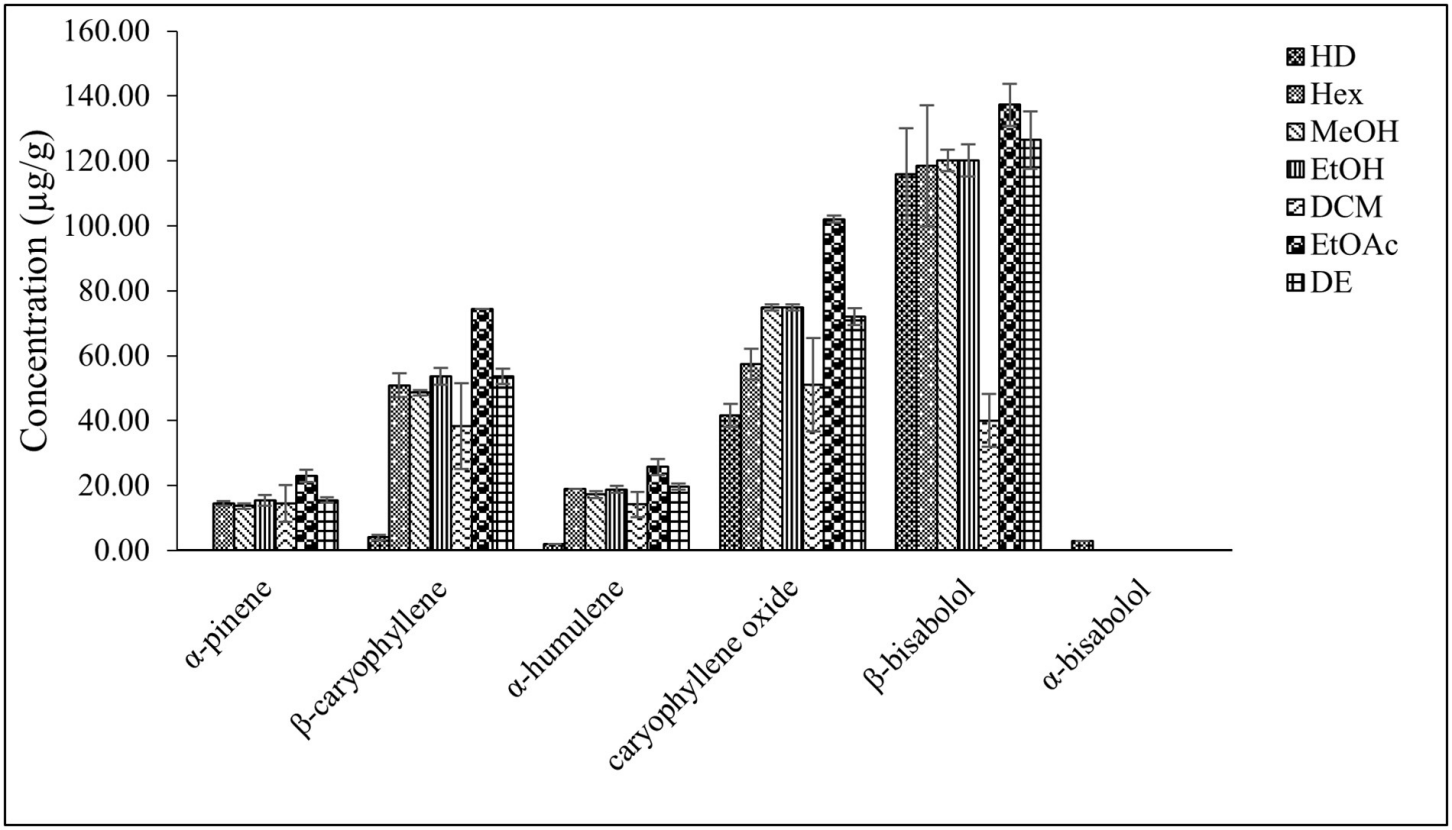
Mean concentration of major terpenoids identified in CGT extracts obtained from hydro-distillation (300 g) and solvent extraction (1 g) of CGT sample replicates (n = 3). Hydro-distillation (HD), hexane (Hex), methanol (MeOH), ethanol (EtOH), dichloromethane (DCM), ethyl acetate (EtOAc) and diethyl ether (DE). Error bars represent standard deviation of terpenoids concentration in replicate samples.

The total ion chromatogram (TIC) in S2 Fig shows terpenoids detected in extracts of hydro-distilled CGT oil against terpenoid standards. Cotton gin trash samples contained 4.0 μg/g β-caryophyllene, 41.5 μg/g caryophyllene oxide, 2.0 μg/g α-humulene, 3.0 μg/g α-bisabolol and 116.0 μg/g β-bisabolol (Fig 4) which was the most abundant terpenoid quantified.

Terpenoid yield by hydro-distillation was compared to yields from use of different organic solvents, to ascertain that the target compounds have been effectively recovered. Extraction of terpenoids from 1 g of CGT samples by use of ethanol (EtOH), methanol (MeOH), diethyl ether (DE), ethyl acetate (EtOAc), hexane (Hex) and dichloromethane (DCM) resulted in the recovery of similar terpenoids extracted by hydro-distillation with the exception of α-bisabolol (Fig 4), which was only identified in hydro-distilled (HD) extracts. Terpenoids quantified in the different organic solvent extracts of CGT samples also show β-bisabolol as the most abundant terpenoid by concentration. The mean concentration of β-bisabolol was calculated to be 118.4 μg/g, 120.2 μg/g, 120.2 μg/g, 40.1 μg/g, 137.3 μg/g and 126.5 μg/g in hexane, methanol, ethanol, dichloromethane, ethyl acetate and diethyl ether extracts, respectively.

The percentage composition of terpenoids in individual organic solvent extracts (S3 Table) was calculated based on the total concentration (μg/g) of major terpenoids quantified in the different extracts. The result presented in the table explains further the proportion of the individual terpenoids against each other in the solvent extracts. Comparing terpenoids recovery from the different organic solvents, it was observed that there was more significant difference (P < 0.001) between solvent extracts for β-bisabolol and caryophyllene oxide than the other terpenoids (P = 0.003, 0.003 and 0.010) (S3 Table).

Total major terpenoids identified were calculated for CGT hydro-distilled and organic solvents extracts and a significant difference (P< 0.001) was observed across the different extracts. S3 Fig shows the total major terpenoids identified in the extracts with Duncan’s multiple range test values indicating similarities between recovery of terpenoids from hydro-distillation (HD) (166.5 μg/g) and dichloromethane (DCM) (158.0 μg/g), as well as similarities between total terpenoids in hexane (Hex) (260.0 μg/g), methanol (MeOH) (274.6 μg/g), ethanol (EtOH) (282.8 μg/g) and diethyl ether (DE) (287.3 μg/g). It is also clear that there was a significant difference between total terpenoids (S3 Fig) extracted using ethyl acetate (362.5 μg/g) and the other extracts.

**Fig 5:**
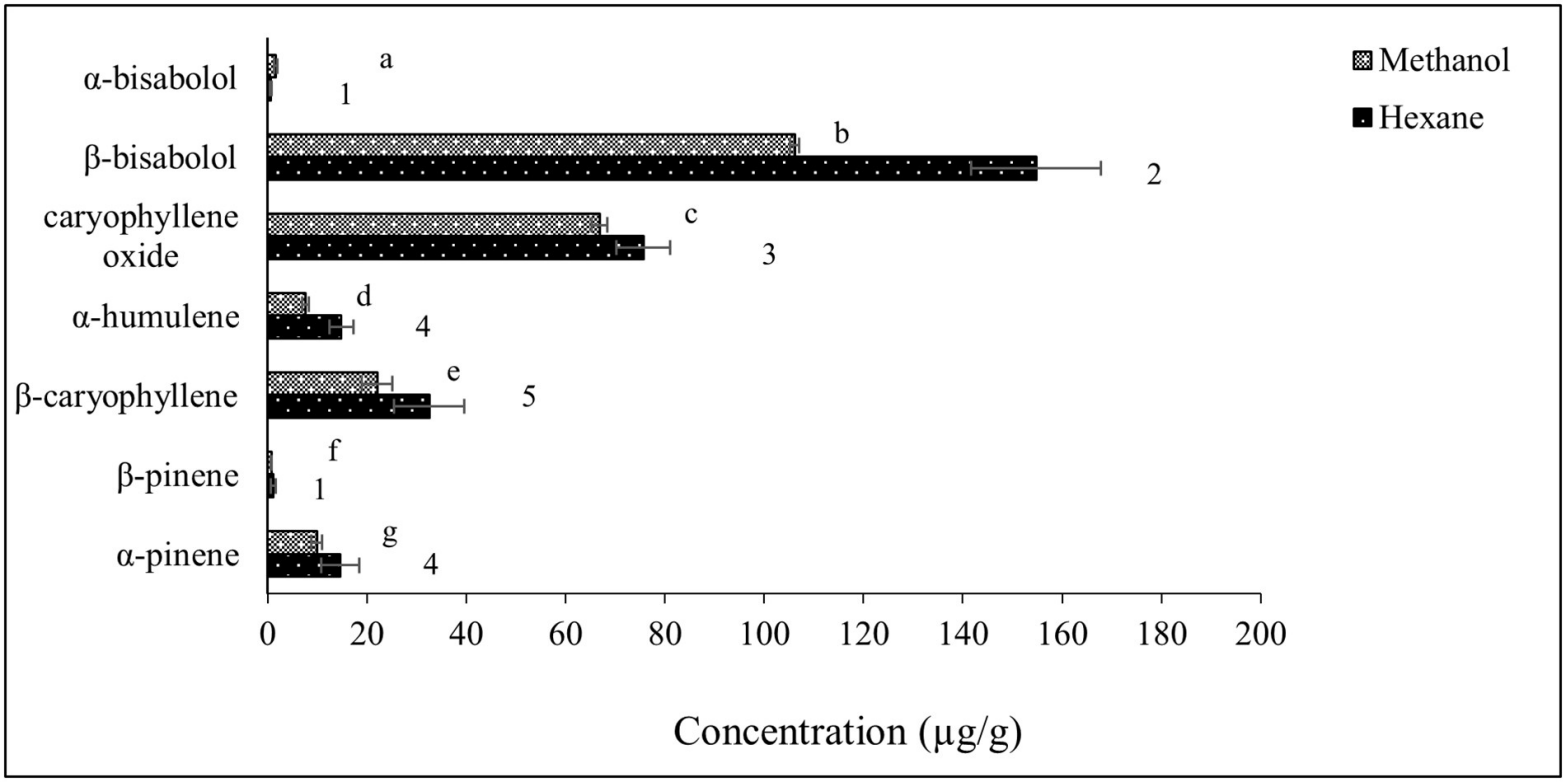
Concentration of terpenoids (μg/g of CGT) detected in methanol and hexane extracts from 40 g CGT samples (n = 3) extracted with 200 mL solvent. Error bars represent standard deviation of terpenoids concentration in replicate samples. Different superscript letters and numerals indicate significant differences (P< 0.05) between terpenes quantified in methanol and hexane extracts of CGT respectively.

Data generated from solvent extraction of volatile compounds from a larger CGT sample size (40 g) show that recovery of volatile terpenoids was higher in hexane extracts compared to methanol extracts (Fig 5). Again, in this extraction procedure, β-bisabolol was the most quantifiable terpenoid in both methanol (106.2 μg/g) and hexane (154.8 μg/g) extracts. With this method of extraction, it was observed that β-pinene and α-bisabolol were detected and quantified in both hexane and methanol extracts from 40 g CGT. These compounds were below the limit of quantification in smaller (1 g) CGT samples extracted with methanol and hexane.

### Quantification of terpenoids in cotton post-harvest trash

Volatiles quantified in hexane extracts from 1 g of different components of the post-harvest trash (PHT) (Table 2), show variation in the concentration of the targeted terpenoids. The leaves and calyx contained more terpenoids compared to the stalks and stems component of the PHT. The highest concentration of β-bisabolol (581 μg/g) was found in the calyx, followed by the leaves (564.8 μg/g), stalk (72.7 μg/g) and stem (18.3 μg/g). On the other hand, the stalk component contained highest concentrations of α-pinene (7.0 μg/g), whereas, the stem component had the lowest composition of all the terpenoids quantified (no α- and β-pinene were detected). Total concentration of target terpenoids quantified in the different components of PHT (Table 2) gives an idea of terpenoid distribution between the different plant parts. It is clear that calyx and leaves contained the most terpenoids as total terpenoid concentrations were 659.1 μg/g and 627.7 μg/g respectively. Stalks and stems contained lower concentration of total terpenoids at 113.0 μg/g and 24.2 μg/g respectively. There was an observed significant difference (P< 0.05) in total terpenoid concentration between the four different components of PHT.

**Table 2:** Mean concentration (μg/g of sample) of terpenoids in different components of post-harvest trash (n = 3) extracted with hexane and determined by GC-FID.

Terpenoids	Concentration (μg/g) in PHT components			
	Calyx	Leaves	Stalks	Stems
α-pinene	2.9 ± 0.1^b^	1.2 ± 0.3^ab^	7.0 ± 1.4^c^	0.0
β-pinene	1.6 ± 0.1^c^	0.3 ± 0.0^a^	0.9 ± 0.1^b^	0.0
β-caryophyllene	16.9 ± 2.8^b^	23.7 ± 2.3^c^	16.0 ± 2.3^b^	3.3 ± 0.1^a^
α-humulene	5.0 ± 2.1^b^	8.9 ± 1.1^c^	5.7 ± 0.5^b^	1.2 ± 0.0^a^
caryophyllene oxide	50.8 ± 8.8^c^	28.8 ± 2.8^b^	10.8 ± 1.4^a^	1.4 ± 0.3^a^
β-bisabolol	582.0 ± 73.6^b^	564.8 ± 57.5^b^	72.7 ± 10.5^a^	18.3 ± 1.6^a^
**Total**	**659.2 ± 87.5**^**a**^	**627.7 ± 64.0**^**a**^	**113.1 ± 16.2**^**b**^	**24.2 ± 2.0**^**c**^

Values presented as mean concentration ± standard deviation (S.D). Different superscript letters indicate significant differences (P< 0.05) of terpenes between the different components of post-harvest trash.

## Discussion

Crop residues can serve as an alternative and cheaper source of biologically active natural products. In this study, the volatile chemical composition of waste materials from cotton ginning was investigated following the hypothesis that bioactive chemicals distributed in parts of the cotton plant could be carried over into cotton gin trash (CGT) during the ginning process. Extraction of bioactive chemicals from plants or plant-based materials is achieved by means of different methods including distillation, use of solvent, maceration, Soxhlet extraction, headspace etc. [24]. Amongst these methods of extraction, hydro-distillation is one of the most common and established essential oil extraction method which recovers several different groups of compounds from plant materials [24,26,31]. This method has proven to be effective for extraction of terpenoids from a variety of plant matrices such as *Eucalyptus citriodora*, citrus, *Matricaria recutita* and their by-products [32–34]. Likewise, results in this study show a variety of terpenoids including monoterpenoids and sesquiterpenoids extracted from CGT by hydro-distillation.

The hydro-distillation process involves temperature and time, which are two important factors [30,35] in essential oil extraction. Elution of monoterpenoids from plant matrix during distillation precedes that of sesquiterpenoids as a result of their smaller molecular mass of 136.24 g/mol and lower boiling points [36]. This was observed in extracts from preliminary distillation of CGT as 100% monoterpenoids were extracted after 6 h of distillation, and, extraction of sesquiterpenoids continued up to 18 h. The duration of 6 h was sufficient for extracting most of the volatile compounds from CGT samples. Composition of terpenoids observed in extracts of hydro-distilled CGT samples corresponds with reported chemical composition of cotton plant parts such as the leaves and whole bolls [14,15,16,37,38]. The observed abundance of sesquiterpenoids compared with monoterpenoids in CGT distilled extracts also correlates with composition of terpenoids in cotton plants [39], with mostly sesquiterpenoids such as β-caryophyllene, caryophyllene oxide, α-humulene, β-farnesene and β-bisabolol reported in the cotton plant. Monoterpenoids such as α- and β-pinene, myrcene and limonene are also present in cotton plants, but their abundance is lower compared to sesquiterpenoids.

Notwithstanding, the different concentration of individual terpenoids identified in CGT extracts from hydro-distillation and solvent extraction, the recovery abundance for all the extracts with the exception of dichloromethane (DCM) was β-bisabolol > caryophyllene oxide > β-caryophyllene > α-humulene > α-pinene or α-bisabolol. In this study, CGT samples were from mature cotton plants and therefore, the composition of volatile terpenoids identified not only gives a clear indication of the chemical profile of the waste material but also provides added knowledge of chemical composition of the cotton plant at full maturity. Terpenoids such as β-caryophyllene, caryophyllene oxide, humulene and β-bisabolol which are constitutive and inducible terpenoids in cotton plant parts including leaves and bracts, are known to play defensive roles against insect attack on the plant. Hence, their continuous synthesis in cotton plant tissues catalysed by the activity of terpene synthases during the period of plant growth, results in carryover of the volatiles into the waste material. The results further reveal that at full maturity, cotton plant terpenoids are mostly comprised of sesquiterpenoids, with the most abundant found to be β-bisabolol, β-caryophyllene and caryophyllene oxide.

The relatively high amount of β-bisabolol (confirmed by NMR spectroscopy) observed in CGT samples is in agreement with Thompson, Baker, *et al*., (1971), Hedin *et al*., (1972) and Elzen, Williams and Vinson (1984) findings which reported the abundance of β-bisabolol in cotton leaves, buds, flowers, as well as being detected in the atmosphere surrounding the cotton plant in the field [15,16,40]. Based on the yields observed for terpenoids in CGT by the two extraction methods, solvent extraction seemed to be the most effective for recovering most of the terpenoids quantified. However, hydro-distillation was effective for enriching β-bisabolol from CGT despite that the total concentration of terpenoids quantified was less than that obtained with organic solvent extraction. This therefore indicates that hydro-distillation could be a better approach in extracting the sesquiterpenoid β-bisabolol. Moreover, considering cost effectiveness and processing of large quantities at a time, hydro-distillation could be the most viable method of recovering the terpenoids from CGT. This assumption is supported by the knowledge that distillation is the preferred and commonly employed method of extracting essential oils from plants on a commercial scale, for example in the tea tree industry [41,42].

Specific terpenoids are concentrated in different parts of the cotton plant at different proportions. Therefore, the concentration of terpenoids identified in the different components of PHT in this study, can be linked to the original distribution of the volatiles in the cotton plant. Terpenoids found in parts of the cotton plant are either stored in that part of the plant or synthesised at the time of need indicating the special functions of these terpenoids in their locations in the plant [43]. In cotton plant, terpenoids are reported to be synthesised and stored in epidermal glands (trichomes) present on different parts of the cotton plant [44–48]. The density of trichomes on the aerial part of the plant is related to terpenoid synthesis in that part of the plant [49–52], including cotton [19]. Trichome density and terpenoid production in leaves of cotton plant are mostly reported, with less emphasis on the other parts of the plant. In their study, Opitz, Kunert and Gershenzon (2008) reported the increased production of terpenoids as trichome density increased during herbivore attack on cotton leaves [19]. Therefore, the concentration of terpenoids observed in the different components of PHT could be attributed to trichome density in the different parts of the plant. This assumption can be supported with the knowledge that terpenoid production in plants including cotton is elevated in response to attack or damage by external factors such as temperature and insect impact [53,54].

Herbivoral activity, which is one of the external factors, is known to target mostly the aerial parts of the cotton plant including leaves, fruits, bolls and flowers, and less frequently other parts such as the stalks and stems [55]. Also, the optimal defence theory (ODT) which proposes that defence compounds are allocated to tissues of a plant which are likely to be targets of herbivoral attack [56], contributes to the assumption that more herbivoral activity on the leaves and calyx of the cotton plant is a possible reason for terpenoid enrichment in these tissues. The distribution of terpenoids observed in PHT components in this study is consistent with the study of Chen *et al*. (2014), which found more sesquiterpenoids in leaves of patchouli than in stems and roots [54]. Also comparing the results of this study to the study of Kasperbauer and Loughrin (2004) which reported the concentration of terpenoids in cotton leaves, concentration of particularly caryophyllene and α-humulene followed a similar pattern as was observed in leaves of PHT [48]. The results of terpenoids distribution in the different components of the PHT also suggests that the leaves or calyx may be the major sources of terpenoids in CGT.

β-Bisabolol, identified as the most abundant terpenoid in CGT, is a sesquiterpenoid alcohol and an isomer of α-bisabolol, a major sesquiterpenoid found in chamomile flowers [57,58] and candeia stems [59]. Although, not as common as its stereoisomer, β-bisabolol has been reported to be one of the major sesquiterpenoids extracted from cotton plants [16,19], and most notably from the cotton bolls [60]. The compound has also been identified in the essential oil of *Santalum album* [61], and in trace amounts in ayou oil from *Aydendron barbeyana Mez* [62]. β-Bisabolol acts as an insect attractant, particularly for boll weevils, and stimulates the production of sex pheromones in the insects, thereby promoting mating between opposite sexes. With limited information on biological properties of β-bisabolol, assumptions may be drawn from the established knowledge of bio-activity of its isomer α-bisabolol [63,64]. It could be deduced that β-bisabolol may have similar biological properties as α-bisabolol which include anti-inflammatory, anti-cancer and antibiotic [65,66]. Considering that our results presented have indicated β-bisabolol being the most abundant volatile in CGT, the waste material could be exploited as a source of this terpenoid.

## Conclusion

In this study, we demonstrate that bio-extractives like terpenoids are present in CGT. Most notably is the high concentration of the sesquiterpenoid alcohol β-bisabolol in extracts from hydro-distillation and organic solvent extraction. Hydro and steam distillations have been commonly used to extract volatiles from plant materials on an industrial scale and they should be suitable to produce essential oil rich in β-bisabolol from CGT. Although not investigated in this study, pH of the extracting solvents maybe exploited to further optimise the recovery of volatile compounds from CGT. The distribution of terpenoids in different components of PHT suggests that these volatile compounds are from specific plant parts and carried over to CGT occurs from harvesting cotton fibres. The presence of the volatiles in CGT and PHT suggests these by-products from cotton industry could be exploited as a source of potentially valuable bioactive compounds.

## Supporting information

S1 Fig Chromatogram of CGT oil. Isolated fraction containing suspected β-bisabolol (black arrow) at UV of 280nm (A) and 210 nm (B) performed by preparative HPLC.
(TIF)

S2 Fig Total ion chromatogram of hydro-distilled CGT oil against terpenoid standards. (TIF)

S3 Fig Total major terpenoids quantified in CGT extracts from hydro-distillation (HD) of 300 g and different solvent extraction of 1 g CGT (n = 3). Hexane (Hex), methanol (MeOH), ethanol (EtOH), dichloromethane (DCM), ethyl acetate (EtOAc) and diethyl ether (DE). Error bars represent standard deviation of terpenoids concentration in replicate samples. Different superscript letters indicate significant differences (P < 0.05).
(TIF)

S1 Table Recovery of CGT oil and volatile terpenoids present in CGT samples from preliminary hydro-distillation. (PDF)

S2 Table Percentage abundance of volatiles in hydro-distilled CGT extracts. (PDF)

S3 Table Mean percentage (n = 3) composition of terpenoids extracted from one gram of CGT samples using different organic solvents. (PDF)
