# Supplementary figures and images for: Chemical volatiles present in cotton gin trash: A by-product of cotton processing
Source: PLoS One. 2019 Sep 18;14(9):e0222146. doi: 10.1371/journal.pone.0222146 (PMC6750886; doi:10.1371/journal.pone.0222146)

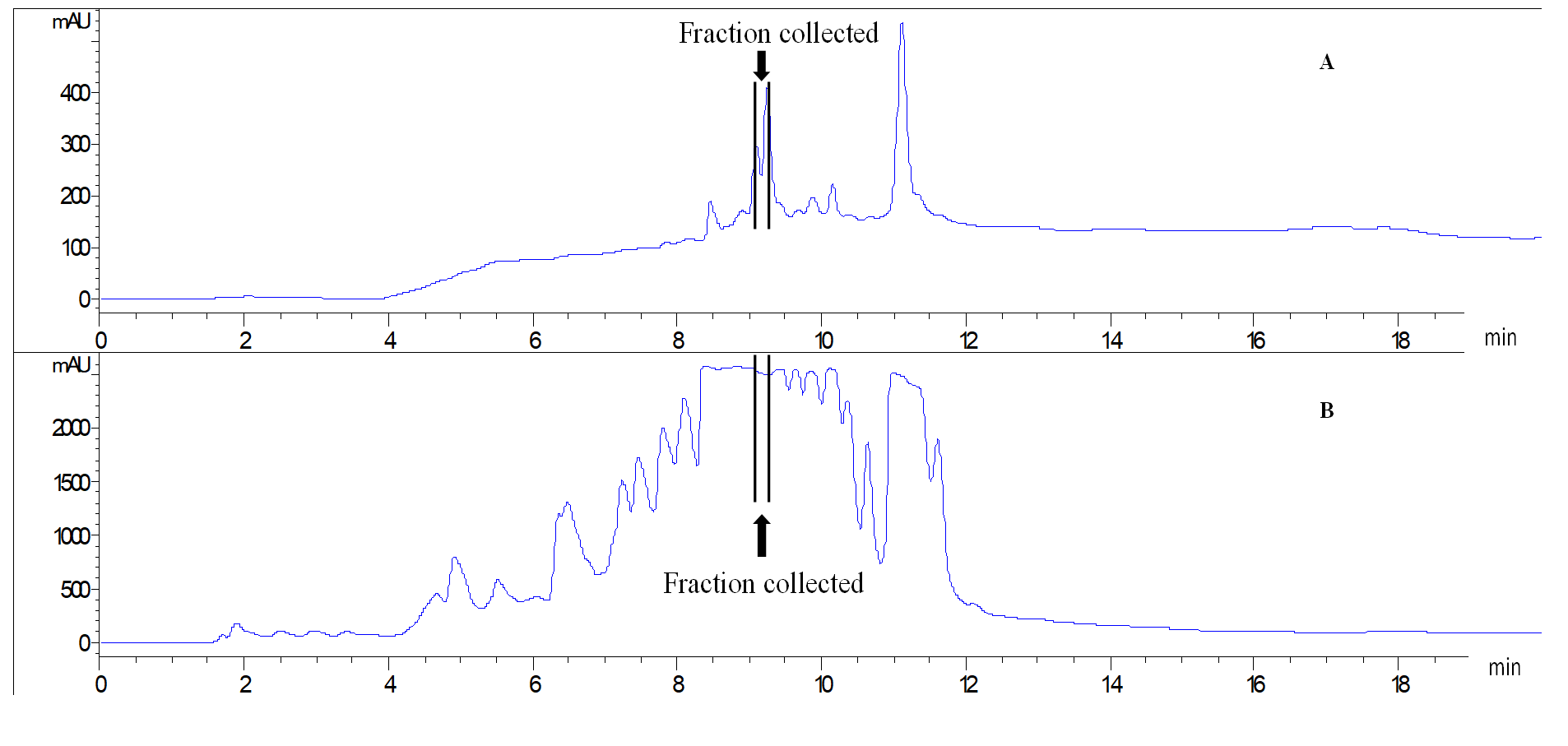

Supplement: S1 Fig — Isolated fraction containing suspected β-bisabolol (black arrow) at UV of 280nm (A) and 210 nm (B) performed by preparative HPLC. (TIF) [file pone.0222146.s001.tif]

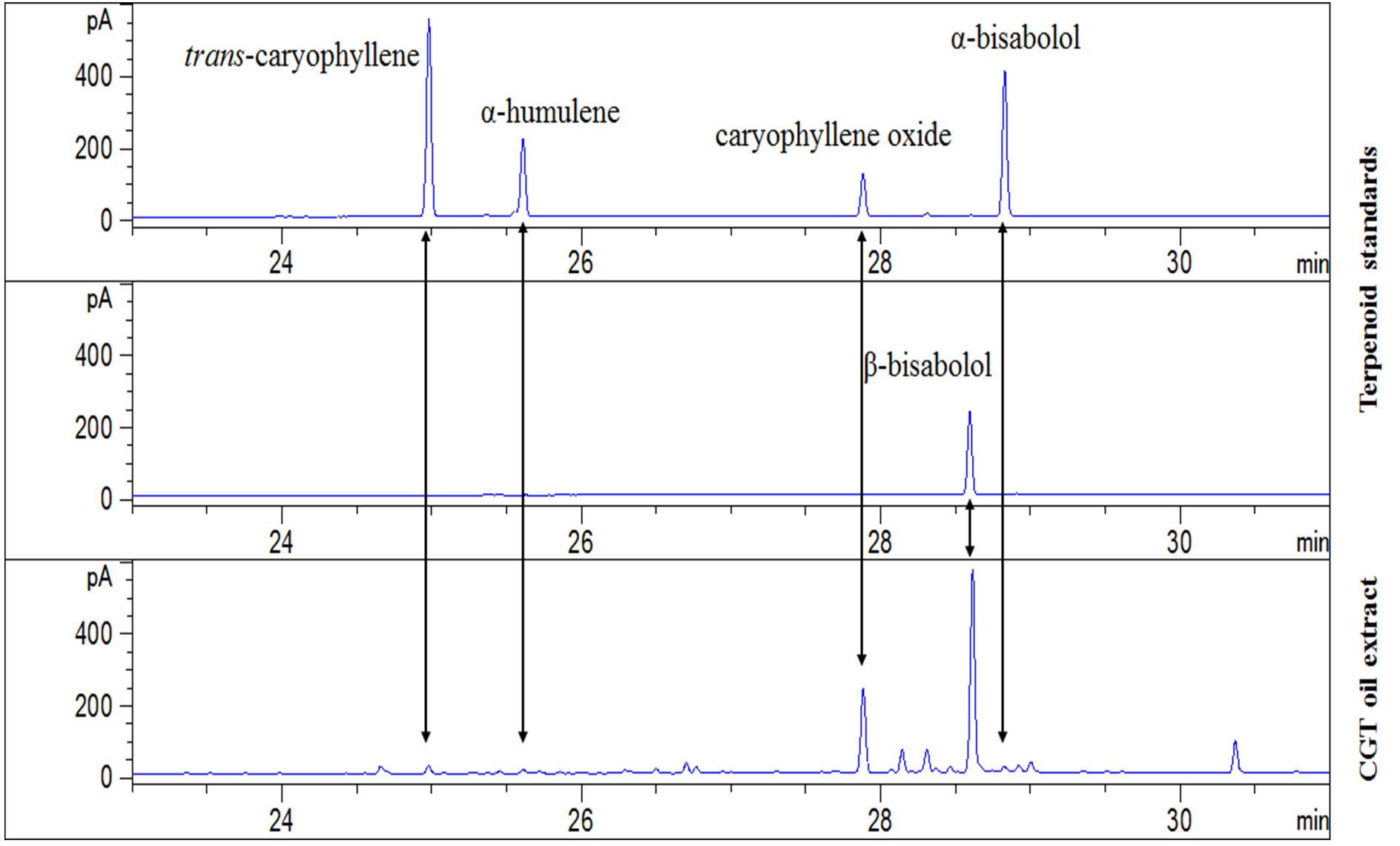

Supplement: S2 Fig — (TIF) [file pone.0222146.s002.tif]

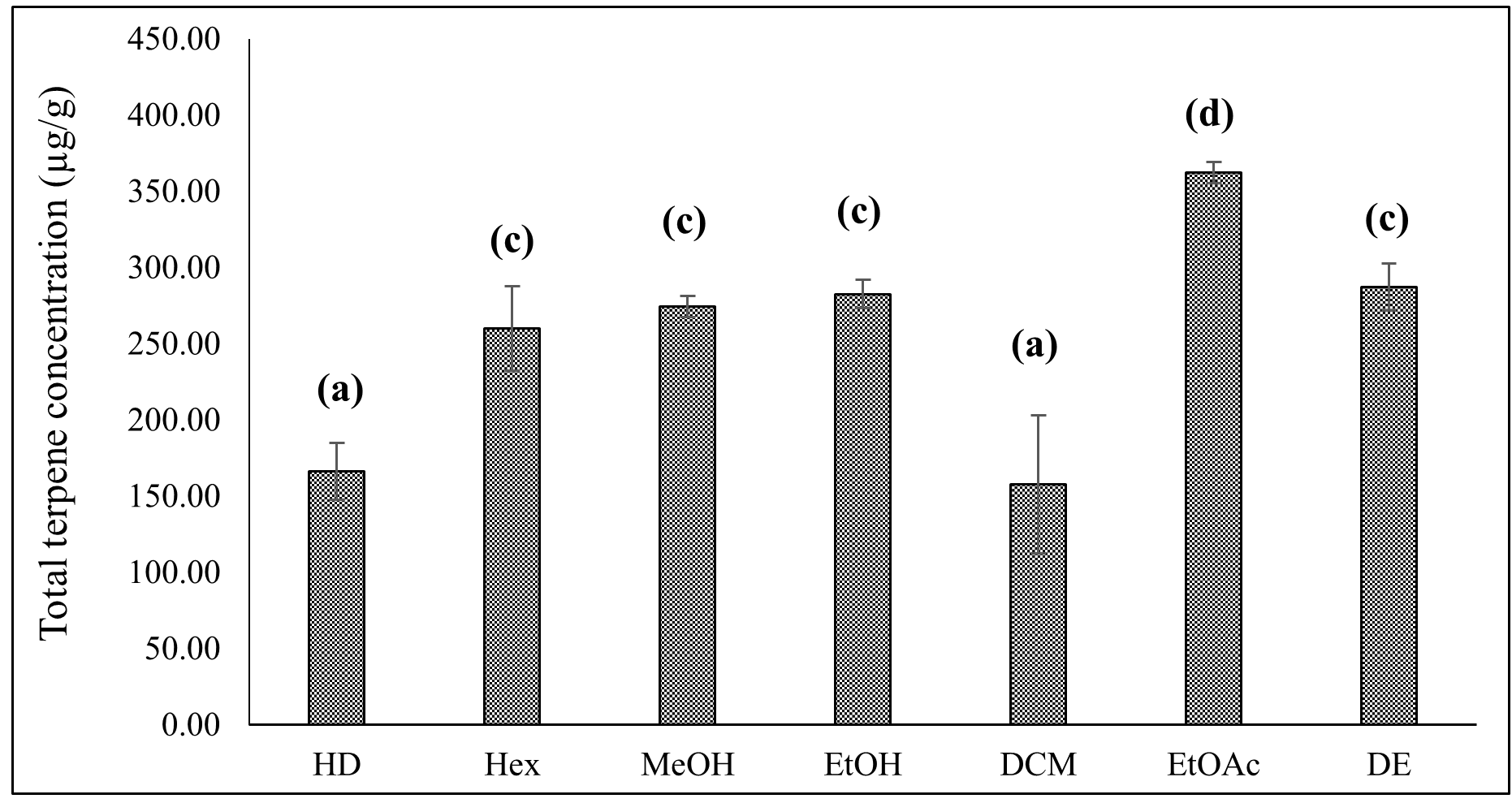

Supplement: S3 Fig — Hexane (Hex), methanol (MeOH), ethanol (EtOH), dichloromethane (DCM), ethyl acetate (EtOAc) and diethyl ether (DE). Error bars represent standard deviation of terpenoids concentration in replicate samples. Different superscript letters indicate significant differences (P < 0.05). (TIF) [file pone.0222146.s003.tif]
