# Supplementary material for: Chemical volatiles present in cotton gin trash: A by-product of cotton processing
Source: PLoS One. 2019 Sep 18;14(9):e0222146. doi: 10.1371/journal.pone.0222146 (PMC6750886; doi:10.1371/journal.pone.0222146)
Supplement: S1 Table — (PDF) [file pone.0222146.s004.pdf]

S1 Table. Recovery of CGT oil and volatile terpenoids present in CGT samples from preliminary hydro-distillation

		**6 h**	**12 h**	**18 h**
**Mass of oil extracted (Percentage)**		260 mg (91.2%)	20 mg (7.0%)	> 10 mg (1.8%)
**Terpenoids identified**	**Match quality (%)**	**% recovery**		
**Monoterpenoids**				
α-pinene	96	100.0	0.0	0.0
myrcene	96	100.0	0.0	0.0
**Sesquiterpenoids**				
α-copaene	99	95.1	4.9	0.0
α-santalene	98	96.6	3.4	0.0
β-caryophyllene	99	96.4	3.1	0.5
β-santalene	96	79.7	17.3	3.0
α-humulene	98	97.1	2.4	0.4
β-farnesene	98	95.4	3.8	0.8
β-bisabolene	65	96.2	3.8	0.0
γ-bisabolene	65	94.7	4.6	0.7
caryophyllene oxide	96	97.6	2.0	0.4
humulene epoxide II	75	95.9	3.6	0.5
gossonorol	65	89.7	9.1	1.2
β-bisabolol	98	89.2	9.2	1.7

*This is a pilot experiment to estimate the hydro-distillation time required for extracting majority of volatile compounds from CGT. No replications have been carried out.
