# Supplementary material for: Chemical volatiles present in cotton gin trash: A by-product of cotton processing
Source: PLoS One. 2019 Sep 18;14(9):e0222146. doi: 10.1371/journal.pone.0222146 (PMC6750886; doi:10.1371/journal.pone.0222146)
Supplement: S2 Table — (PDF) [file pone.0222146.s005.pdf]

S2 Table. Percentage abundance of volatiles identified in hydro-distilled CGT extracts

Volatile compounds	R.T	% abundance
α-pinene	13.23	0.04
myrcene	14.19	0.02
β-pinene	14.33	0.01
(n-)dodecanol	16.02	0.03
6-methyl-3,5-heptadiene-2-one	17.03	0.41
α-campholenal	17.73	0.19
trans-verbenol	18.18	0.17
camphor	18.91	0.09
2-isopropyl-5-oxohexanal	18.57	0.06
9-oxabicyclo[6.1.0]non-3-yne	18.66	0.05
hydroxymethylbenzene	18.70	0.05
safranal	19.36	0.03
ethyl maltol	19.50	0.05
verbenone	19.63	0.07
β-cyclocitral	19.74	0.05
(2E,4E-)dodecadienal	21.30	0.23
2,5-diethylfuran	21.73	0.12
α-copaene	22.59	0.26
α-santalene	23.18	0.19
neryl acetone	23.31	2.86
β-caryophyllene	23.57	2.40
(epi- β-)santalene	23.57	0.31
tetracyclo[5.2.1.0(1,6)]decan-6-ene	23.90	0.30
β-santalene	23.90	0.47
α-curcumene	24.09	0.42
α-humulene	24.19	1.21
β-farnesene	24.33	0.73
β-bisabolene	24.50	0.42
2,3-dihydro-1,3,3-trimethyl-1H-indole	24.56	0.33
((E)-γ-)bisabolene	24.34	0.68
2,3-dihydro-1,1,3-trimethyl-1H-indene	24.70	0.15
5-amino-1-ethylpyrazole	24.82	0.36
2,4-dimethyl-quinoline,	24.92	2.01
(E-)nerolidol	25.16	1.21
spiro(Demirel et al.)decane	25.33	2.73
4,8-dimethyl-2-(2-methyl-1-propenyl)-1-	25.40	1.89
3,7-dimethyl-8-methoxypyrimido	25.46	0.65
cubebol	25.80	0.20
1,1,6-trimethyl-calacorene	25.87	0.32
2-chloro-2,3,3-bicyclo[2.2.1]heptane	25.93	0.09
6,6-dimethyl-3-bicyclo(Jaenson et al.)heptane	26.23	0.42
caryophyllene oxide	26.45	15.51
1-(2-ethoxyphenyl)acetone	26.45	1.77
santolina triene	26.64	0.67

gossonorol	26.76	5.68
humulene epoxide	26.87	5.28
β-bisabolol	27.25	28.92
5-butyl-2-methoxy-3-methylpyrazine	27.52	0.97
α-agarofuran	28.58	0.81
3-(1,1-dimethylethyl)-1,2-naphthalene	28.71	0.24
3,7-dimethyl-1,6-octadiene	28.82	0.06
2-pentadecanone	29.06	7.74
(5-hydroxy-cis-)calamenene	29.35	5.11
di-isodecyl phthalate	29.70	0.29
4,5,6,7-tetramethyl-2H-isoindole	29.78	1.28
farnesyl acetone	30.17	2.34
phytol	30.38	0.48
n-hexadecanoic acid	30.87	0.22
farnesol	30.98	0.35
