# Supplementary material for: Chemical volatiles present in cotton gin trash: A by-product of cotton processing
Source: PLoS One. 2019 Sep 18;14(9):e0222146. doi: 10.1371/journal.pone.0222146 (PMC6750886; doi:10.1371/journal.pone.0222146)
Supplement: S3 Table — (PDF) [file pone.0222146.s006.pdf]

S3 Table. Mean percentage (n=3) composition of terpenoids extracted from one gram of CGT samples using different organic solvents

	**α-pinene**	**β-caryophyllene**	**α-humulene**	**caryophyllene oxide**	**β-bisabolol**
**Hex**	5.5 ± 0.3^{ab}	19.6 ± 0.7^{ab}	7.3 ± 0.8^{a}	22.1 ± 0.5^{a}	45.6 ± 2.3^{c}
**MeOH**	4.9 ± 0.2^{a}	17.7 ± 0.1^{a}	6.3 ± 0.2^{a}	27.2 ± 0.3^{cd}	43.8 ± 0.1^{c}
**EtOH**	5.5 ± 0.4^{ab}	18.9 ± 0.3^{ab}	6.7 ± 0.2^{a}	26.5 ± 1.2^{c}	42.5 ± 0.3^{c}
**DCM**	9.1 ± 1.0^{c}	24.3 ± 1.5^{c}	9.0 ± 0.1^{b}	32.3 ± 0.1^{e}	25.3 ± 2.2^{a}
**EtOAc**	6.4 ± 0.4^{b}	20.5 ± 0.4^{b}	7.1 ± 0.8^{a}	28.2 ± 0.3^{d}	37.9 ± 1.1^{b}
**DE**	5.4 ± 0.1^{ab}	18.7 ± 0.2^{ab}	6.8 ± 0.1^{a}	25.1 ± 0.5^{b}	44.0 ± 0.7^{c}
P	0.003	0.003	0.010	<0.001	<0.001
**HD**		2.4 ± 0.2^{1}	1.3 ± 0.1^{1}	24.5 ± 0.5^{2}	69.8 ± 0.5^{3}

Values are mean percentage composition ± standard deviation of terpenoids quantified. Different superscript letters indicate significant differences ($P < 0.05$) between solvents for the same terpenoid, whereas, different superscript numbers indicate significant difference between terpenoids extracted by hydro-distillation. Percentage composition calculated based on total concentration (µg/g) of major terpenoids identified and quantified in the different organic solvent extracts of CGT.
